# Louisville Marine Hospital

**Published:** 1840-10

**Authors:** 


### LOUISVILLE MARINE HOSPITAL.

It is pretty universally conceded, we believe, that Clinical practice cannot be rendered of much avail to a large medical class, where the patients are examined and prescribed for in the ordinary wards of a hospital. But few, comparatively, of such a class, can see either the patient or the medical attendant, and all interest is soon lost in the case where the ear alone is addressed and the symptoms cannot be noted. Impressed with this disadvantage, and regarding the Hospital as the right arm of the Medical Institute, the Faculty determined, last winter, on erecting a Lecture and Operating Room in connexion with the main building. This room is now in progress and will be in readiness for the class by the last of October. It is placed a few feet south of the old building, of which it will form a wing, being connected with it by a gallery, along which the patients, either for medical or surgical treatment, will be carried on their beds from the wards without exposure or inconvenience. The room will the be capable of seating four hundred persons, and being arranged in form of an amphitheatre, with sky-lights, will afford to each student a full view of the patient while undergoing examinations or operations. In this arrangement, the well-founded objection against the usefulness of hospitals to large classes of medical students, that they can neither see the sick, nor distinctly hear the prescriptions for them, will be wholly obviated. The Institute will be fully in possession of this most important element of medical instruction.— A sufficient number of patients is always found in the medical and surgical wards to give interest and value to clinical lectures. Y.

